# Web-Fingered Child, with Extra Fingers and Toes

**Published:** 1864-05

**Authors:** O. L. Abbey

**Affiliations:** Wattsburg


WEB-FINGERED CHILD, WITH EXTRA FINGERS AND TOES.

WATTSBURG, April 29, 1864.

Editor Buffalo Medical and Surgical Journal :

If you think the following worthy of notice, you may publish it:—Was called to see Mrs. D—— April 10th, who gave birth in a natural and easy labor to a male child, that had five fingers on each hand, and six toes on each foot, was also web-fingered; in other ways natural. I removed the extra fingers and cut the others apart, which was all it would bear at that time. Child and mother both did well.

Truly yours, O. L. ABBEY.

